# Solvate‐Induced Semiconductor to Metal Transition: Flat 1∞ [Bi^1−^] Zigzag Chains in Metallic KBi⋅NH_3_ versus 1∞ [Bi^1−^] Helices in Semiconducting KBi

**DOI:** 10.1002/anie.201915735

**Published:** 2020-03-02

**Authors:** Kerstin Mayer, Jasmin V. Dums, Christian B. Benda, Wilhelm Klein, Thomas F. Fässler

**Affiliations:** ^1^ Technische Universität München Institute of Inorganic Chemistry Lichtenbergstrasse 4 Garching bei München 85747 Germany

**Keywords:** bismuth, structure elucidation, metals, semiconductors, Zintl Phase

## Abstract

Polymeric 1∞ [Bi]^−^ in KBi⋅NH_3_ has planar zigzag chains with two‐connected Bi atoms and metallic properties, whereas KBi, which has helical chains of Bi atoms, is semiconducting. The isomerization of the Bi chain is induced by solvate molecules. In the novel layered solvate structure uncharged 2∞ [KBi] layers are separated by intercalated NH_3_ molecules. These layers are a structural excerpt of the iso(valence)electronic CaSi, whose metallic properties arise from the planarity of the zigzag chain of Si atoms. Computational studies support this view, they show an anisotropic metallic behavior along the Bi chain. Electron delocalization is also found in the new cyclic anion [Bi_6_]^4−^ isolated in K_2_[K(18‐crown‐6)]_2_[Bi_6_]⋅9 NH_3_. Although [Bi_6_]^4−^ should exhibit one localized double bond, electron delocalization is observed in analogy to the lighter homologues [P_6_]^4−^ and [As_6_]^4−^. Both compounds were characterized by single‐crystal X‐ray structure determination.

## Graphical abstract

**Bismuth plan**: The polymeric anion ▪${{{\hfill 1\atop \hfill \infty }}}$▪[Bi]^−^ has planar zigzag chains with two‐connected Bi atoms and metallic properties, whereas KBi, which has helical chains of Bi atoms, is semiconducting. The isomerization of the Bi chain is induced by solvate molecules. Electron delocalization is also found in the new cyclic anion [Bi_6_]^4−^ isolated in K_2_[K(18‐crown‐6)]_2_[Bi_6_]⋅9 NH_3_.

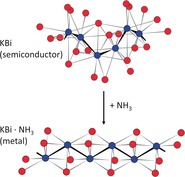

The pseudo‐element concept according to Zintl and Klemm is a widely used method to explain structures of electron‐precise Zintl phases as it expresses the tendency of bond localization in intermetallic compounds.^1^ Originally applied only for semiconductors, this concept was continuously expanded to other systems, though the implementation at the border to typical metals still remains difficult.^2^ Zintl phases are intermetallic compounds that follow a salt‐like description that are electron precise and in general semiconductors. The Zintl phase CaSi is a text book example of an electron‐precise Zintl phase with sulfur analogue two‐connected “Si^2−^” anions, which however, because of the planar conformation of the polyanion, is metallic. Experimental electron density measurements show that Ca orbital contributions play an important role for the planarity of the Si zigzag chain (in contrast to the expected helical chain) enforcing the metallic properties.^3^ However, the Zintl concept does not allow specific conformers of polyanions to be predicted since the role of the counter ions is neglected at first glance.^3^, ^4^ An exemplary variation of structure–property relations already arises in the elements of the pnictogen (*Pn*) group, which embraces the whole spectrum of element types, reaching from the clear non‐metal nitrogen through phosphorous that adapts non‐metallic and metallic modifications, to the semimetals arsenic and antimony and finally to bismuth that is best described as semi‐metal with zero band gap.^5^ The various allotropes of the heavier elements exhibit a huge diversity in their structures, and their affinity to form localized bonds is mirrored in the different cages of polycyclic polyanions, with [*Pn*_4_]^2−^, [*Pn*_7_]^3−^, [*Pn*_11_]^3−^, or [*Pn*_14_]^4−^ as typical examples (*Pn*=P, As, Sb, Bi).^6^ Bismuth, the heaviest homologue, additionally forms stable polycations, such as [Bi_9_]^5+^,^7^ [Bi_8_]^2+^,^8^ or [Bi_5_]^3+[9]^ that are electronically best described as Wade clusters.^10^ These polycations build heterometallic clusters with noble metals in subhalogenide compounds.^11^ As polyanionic species, Bi tends to appear as chain fragments. In solution such polyanions are accessible through extraction of the compounds K_5_Bi_4_, K_3_Bi_2_, or KBi with appropriate solvents. The anions of these binary solids likewise consist of covalently connected Bi atoms. The smallest anion has been described as a double‐bonded Bi dumbbell [Bi=Bi]^2− [12]^ in K_3_Bi_2_, while the planar zigzag tetramer [Bi_4_]^4− [13]^ in K_5_Bi_4_ features a delocalized double bond. According to the 8‐N rule both compounds contain an additional free electron which most likely is delocalized. In KBi the polymeric 1∞ [Bi]^−^ anion complies the Zintl–Klemm pseudo‐element concept by forming a helical chain structure analogous to that of gray selenium or tellurium.^14^

Dissolving the binary solids in suitable solvents, such as ethylenediamine or liquid ammonia, results in deeply colored solutions. However, as a solvate structure solely the square‐planar ring [Bi_4_]^2− [15]^ has been known for a long time, isolated from an ethylenediamine solution of K_5_Bi_4_ in the presence of cryptand[2.2.2]. Later, the dimer [Bi_2_]^2−[12]^ was obtained from solutions of K_3_Bi_2_. Other established pnictides such as the polycyclic cages [*Pn*_7_]^3−^ and [*Pn*_11_]^3−^ (*Pn*=P, As, Sb)^6^, ^16^ followed a few years later; whereas [Bi_7_]^3−^ was obtained from a solution of K_5_Bi_4_ in pyridine,^17^ the [Bi_11_]^3−^ ion was not accessible by a direct route from an exclusively Bi‐containing solid, but was formed upon the decomposition of [GaBi_3_]^2−^ in pyridine.18a Whereas polybismuthide anions appear in rather complex polyanions, such as [(Bi_6_)Zn_3_(TlBi_5_)]^4−^ as ligands,18b bare Bi anions are less frequent. Another example of a polybismuthide is [Bi_4_]^6−^ with its four‐atomic planar zigzag chain, which is the Zintl anion with the highest charge per atom in solution and was obtained from solutions of KBi in liquid ammonia.^19^ These solvate structures already suggest that the bismuthides can easily rearrange in solution, which is mirrored by the dissolution and recrystallization of the Laves phase KBi_2_ from ethylenediamine. Applying a formal charge transfer, the Laves phase KBi_2_ contains the polyanion 3∞ [Bi_2_]^−^ which is isoelectronic to the “monomer” cyclo‐[Bi_4_]^2−^, present in solution.15b

Herein, we report on two new bismuth anions which appear in the transition from electron‐precise Zintl anions to metallic polymers. The flat zigzag chain in KBi⋅NH_3_ (**1**) represents a variation of the Bi substructure in the solvent‐free KBi and is structurally related to the iso(valence)electronic, metallic CaSi. The cyclic anion [Bi_6_]^4−^ in K_2_[K(18‐crown‐6)]_2_[Bi_6_]⋅9 NH_3_ (**2**) is a missing link in the series of Zintl ions of the lighter homologues P and As.

KBi⋅NH_3_ (**1**) contains a polymeric planar 1∞ [Bi]^−^ zigzag chain (Figure 1 a) and was formed in several reaction mixtures in liquid ammonia containing different bismuthide precursors: from the reaction of K_5_Bi_4_ in the presence of Ph_2_Zn and 18‐crown‐6 as well as from a mixture of K_3_Bi_2_ and K_4_Sn_9_. As the other reactants do not play an obvious role in the reactions, we suggest an equilibrium of different bismuthide species in liquid ammonia. KBi⋅NH_3_ (**1**) crystallizes as gray metallic plates in the monoclinic space group *Cm* (no. 8). The Bi−Bi separation of 3.0758(4) Å in the planar zigzag chain of the anion is within the range of typical Bi−Bi single bonds and comparable to the distance in Bi metal with 3.072 Å.^5^ The structure of the chain compares to the planar zigzag arrangement of Bi_4_ in the binary K_5_Bi_4_ 13 and in the solvate K_6_[Bi_4_]⋅8 NH_3_.^19^ In K_5_Bi_4_ the Bi−Bi separations of 2.998 Å and 3.046 Å, respectively, are slightly shorter than in compound **1**, owing to the delocalized double bond in [Bi=Bi−Bi−Bi]^4−^. In K_6_[Bi_4_](NH_3_)_8,_ the *exo* bond of [Bi−Bi−Bi−Bi]^6−^ of 3.083 Å is in the range of the polymer bond length, the central Bi−Bi bonds, however, are significantly longer (3.206 Å). The Bi‐Bi‐Bi bond angle of 116° in **1** is significantly larger than the ones in K_5_Bi_4_ (106°) and K_6_[Bi_4_](NH_3_)_8_ (109°).

**Figure 1:**
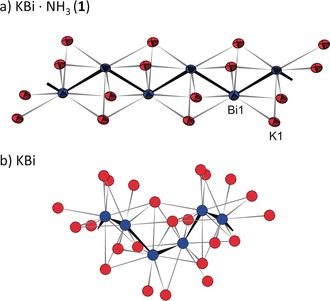
a) Section of the 1∞ [Bi]^−^ zigzag chain in the solvate KBi⋅NH_3_ (**1**) with coordinating K atoms.^26^ Bi−Bi bond 3.0758(4) Å, Bi‐Bi‐Bi angle 116°. The thermal ellipsoids are set at 90 % probability; b) section of the helical chain of KBi with surrounding K atoms. Bi−Bi separations 3.06–3.07 Å.^14^ Bi blue, K red.

Intriguingly, the composition of compound **1** differs only by a single ammonia molecule per formula unit from the one of the binary solid KBi. Both, KBi⋅NH_3_ (**1**) and KBi, feature polymeric 1∞ [Bi]^−^ chains but with different planar zigzag and helical conformation, respectively.^14^ The different chain types are accompanied by a disparate coordination sphere of the K cations. In **1** each Bi atom is in the center of a trigonal prism of six K atoms, whereas the coordination numbers of the Bi atoms in KBi are 7 and 8, respectively (Figure 1 and Figure 2 a).

**Figure 2:**
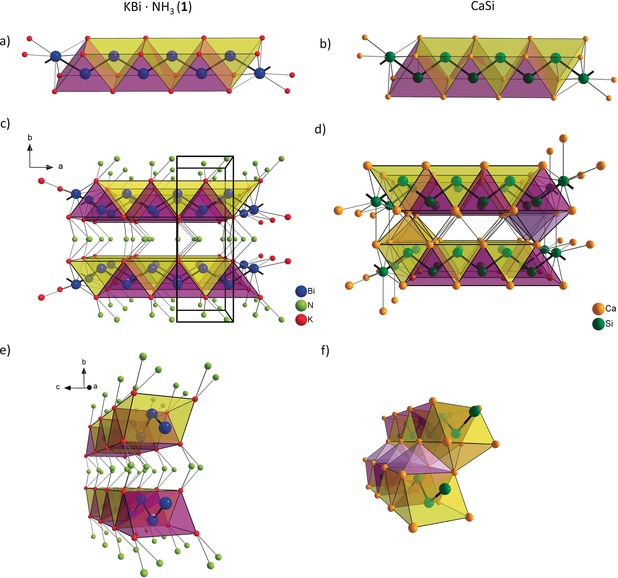
Sections of the crystal structures of a) KBi⋅NH_3_ (**1**); each Bi atom is in the center of a trigonal prism formed by K atoms; b) a similar strand in CaSi; c) extended view of **1**, revealing that the Bi chain is running parallel to the *ab* plane of the unit cell, the 2∞ [KBi] layers are separated by ammonia molecules; d) analogous view of CaSi illustrating two layers; e) in compound **1** the layers are separated by ammonia molecules, and are stacked in direction of the *b* axis; f) the layers of CaSi are connected with Ca atoms of the next layer, hence they are also shifted. Bi blue, N green, K red, Ca orange, and Si dark green. The trigonal prisms are highlighted in yellow and violet, the additional capping in CaSi is shown in light violet and light orange.

The 1∞ [Bi]^−^ chain in **1** is oriented in direction of the crystallographic *a* axis and runs parallel to the *ab* plane. The K_6_ trigonal prisms around each Bi atom share a trigonal face along the crystallographic *c* axis, resulting in 2∞ [KBi] layers consisting of condensed trigonal prisms (Figure 2 a). These layers are separated in direction of the crystallographic *b* axis by intercalated ammonia molecules, which coordinate to the K cations (Figure 2 c).

This structural element is reminiscent of the metallic Zintl phase CaSi.^20^ Like the Bi atoms in compound **1**, the Si atoms in CaSi form a planar zigzag chain surrounded by trigonal prismatic coordination polyhedra of Ca atoms, forming layers of 2∞ [CaSi] (Figure 2 b). However, whereas the layers of compound **1** are separated, the prisms in CaSi are additionally capped by a Ca atom of the next layer (Figure 2 e,f). The metallic CaSi is iso(valence)electronic to the semiconductor KBi, which, as mentioned before, contains helical chains. This discrepancy might be explained by an effect of a partly covalent bonding of the Ca atoms to the delocalized π system of the Si zigzag chains.^3^

In this light, the 2∞ [KBi] layers consisting of condensed trigonal prisms of K atoms in **1** can be comprehended as an excerpt of the CaSi structure with layers separated by ammonia molecules and most probably similar electronic features (Figure 2 c,d). The two existing conformers of KBi might serve as models for the influence of the alkali metal polyanion contact on the shape of the polyanion.

Since the two structures differ only by one NH_3_ molecule the influence on the electronic structure was investigated, and both structures were optimized at a PBE0/TZVP and PBE/TZVP level of theory. Two different functionals were used to confirm the metallic or non‐metallic behavior in a borderline case. PBE0 tends to overestimate the band gap, whereas PBE rather underestimates it. For the two functionals the binary solid KBi appears semiconducting with a band gap of 1.5 eV and 0.5 eV, respectively (Figure 3 a). By contrast, compound **1** is metallic for both functionals (Figure 3 b). The band structure of **1**—shown are paths in the Brillouin zone along the Bi chain and perpendicular to the chain (Figure 3 c)—reveals that the metallic character arises along the direction of the chain and perpendicular to the chain along the *c* axis, whereas a band gap exists in direction of *b* perpendicular to the layers, turning this compound into an anisotropic metal. An orbital projected density of states (DOS) is shown in the Supporting Information indicating a major participation of the p orbitals, in particular of Bi*‐*p_*z*_, that appear close to the Fermi level. Additionally, a band projected density matrix was calculated to point out the p_*z*_ participation of the Bi atoms (Figure 3 d).

**Figure 3:**
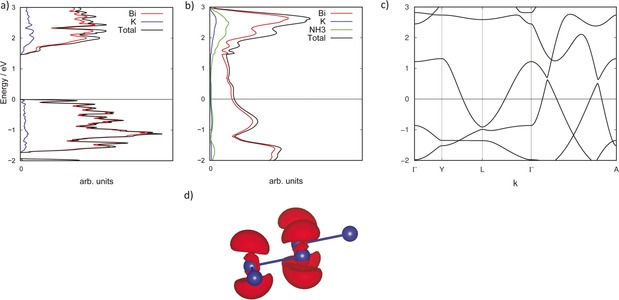
Density of States (DOS) and band structure in the energy range of −2 to 3 eV (0 eV is set for the Fermi level and marked by a horizontal line) based on the PBE0 functional. a) DOS of KBi, atom projection is shown in red for Bi and blue for K. b) DOS of KBi⋅NH_3_ (**1**). Atom projection is shown in red for Bi, blue for K, and green for NH_3_. c) Band structure of **1**. The path Γ→A corresponds to the *a* direction along the Bi chain, the paths Γ→Y and Y→L to the *b* and *c* direction, respectively. A representation of the Brillouin zone and detailed information on the band structures are given in the Supporting Information; d) Band projected electron density of the energetically highest band located below the Fermi level showing a p_*z*_ orbital type character. Bi is shown as blue spheres, the electron density with isovalue 0.006 is shown in red.

The transition from semiconducting KBi to (anisotropic) metallic KBi⋅NH_3_ could be interpreted as a “solvate‐induced metallization”. A similar influence of the intercalation of solvent molecules on the electronic structure was recently reported for the superconducting properties of *A_x_*(NH_2_)_*y*_(NH_3_)_*z*_Fe_2_Se_2_ compared to that of FeSe. In this example lithium ions, lithium amide, and ammonia act as spacer layer between FeSe layers in contrast to the three‐dimensional structure of FeSe along with electron doping.^21^

Intrigued by the question, if it is possible to produce **1** as bulk material by just directly transferring KBi into **1** we stored KBi for two months in liquid ammonia, resulting in a blueish purple suspension. After removing the liquid ammonia, the resulting black powder consists of KBi and KBi_2_ according to X‐ray diffraction. However, **1** is rather temperature sensitive and decomposes after removal of liquid ammonia, as we observed that isolated crystals degas during warming to room temperature.

K_2_[K(18‐crown‐6)]_2_[Bi_6_]⋅9 NH_3_ (**2**) can be obtained either from a solution of K_5_Bi_4_ and Mesnacnac_2_Zn_2_ (Mesnacnac=*N*,*N′*‐bis(2,4,6‐trimethylphenyl)‐β‐diketiminate), or from a mixture of K_3_Bi_2_ with K_4_Ge_9_ in liquid ammonia. In the presence of 18‐crown‐6, a fourfold negatively charged six‐membered [Bi_6_]^4−^ ring is formed. Compound **2** crystallizes as silverish needles in the orthorhombic space group *Pmn*2_1_ (no. 31). The anion [*Pn*_6_]^4−^ is already known for the lighter homologues P and As.^22^, ^23^ The six‐membered Bi_6_ ring in **2** consists of four crystallographically independent Bi atoms and exhibits a chair conformation, with Bi−Bi separations between 2.954(2) and 2.980(2) Å (Figure 4) and bond angles between 116.4(1)° and 119.8(1)°. The dihedral angles of the triangle including Bi1 and Bi4 and the planar square Bi2‐Bi3‐Bi3′‐Bi2′ are 19.1(1)° and 23.9(1)°, respectively, corresponding to a strongly flattened and, thus, weakly defined chair conformation of this six‐membered ring (Figure 4 a).

**Figure 4:**
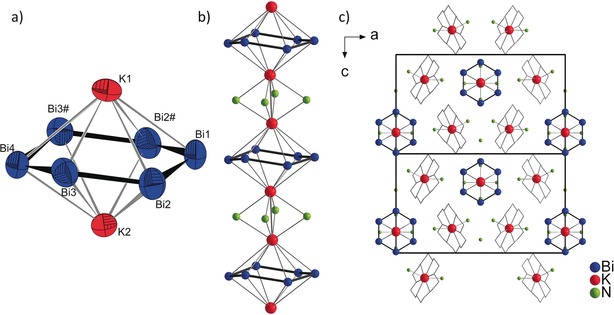
a) Structure of [Bi_6_]^4−^. The interatomic distances [Å]: Bi1−Bi2 2.954(2), Bi2−Bi3 2.966(1), and Bi3−Bi4 2.980(2). Thermal ellipsoids are set at 70 % probability; b) the Bi anion forms an infinite strand [K_2_Bi_6_(NH_3_)_4_]^2−^ with two K atoms and four NH_3_ molecules; c) two extended unit cells of compound **2**. Bi blue, K red, N green. 18‐Crown‐6 is drawn schematically.

According to the 8‐N rule, a four‐fold negatively charged Bi_6_ ring would require one double bond. Kraus et al. discussed the electronic situation of the analogous [As_6_]^4−^ anion: although the free anion should exhibit a boat conformation with a partial localization of double bonds at the shorter As–As separations, the topological analysis of the “Rb_4_As_6_” fragment revealed an even electron distribution over all As atoms.^24^ As a result of the strong structural analogy, including the ion packing, the same model may also apply for the [Bi_6_]^4−^unit. The alkali metal atoms in **2** coordinate to both sides of the coplanar [Bi_6_]^4−^ rings (Figure 4 a). The 1∞ {K_2_[Bi_6_]^2−^} units are connected via four ammonia molecules which coordinate to the potassium atoms, assembling infinite strands of 1∞ {[K_2_[Bi_6_](NH_3_)_4_]^2−^} (Figure 4 b). The additional two potassium cations are sequestered by 18‐crown‐6 molecules, and their coordination sphere is completed by two additional NH_3_ molecules each. Each [K_2_[Bi_6_](NH_3_)_4_]^2−^ strand is surrounded by six K atoms, sequestered by 18‐crown‐6 molecules, resulting in a hexagonal rod packing in direction of the crystallographic *b* axis (Figure 4 c).

An investigation of the behavior of bismuthides in solution reveals the diverse reactivity of these anions. The stepwise fragmentation of structural units from binary solids to give solvated polyanions allows for a better understanding of the Zintl concept. The polymeric zigzag chain 1∞ [Bi]^−^ of compound **1** is a structural motif which has not yet been observed for bismuthides in compounds that are formed in solution. It has the same composition as the semiconductor KBi despite of one additional solvent molecule per formula unit, but its flat zigzag chain is more related to the structure in the iso(valence)electronic compound CaSi revealing metallic properties. From this observation we assume that the coordination of the Bi atoms by K in the shape of condensed trigonal prisms similar to that of the Si atoms by Ca in CaSi is the structure‐determining parameter and, thus, is responsible for the electronic properties. In addition, quantum chemical calculations indicate that this relation to CaSi is well founded, as they reveal compound **1** as an (anisotropic) metal. An inspection of all known bismuthide solvate structures suggests that the coordination by K atoms seems to be crucial for the distinct geometry. A planar zigzag chain is adopted only in case of a strongly ordered K coordination sphere, as seen in compound **1** and in the Bi_4_ chain fragments in K_6_[Bi_4_]⋅8 NH_3_.^19^ A sequestering of the K atoms by a cryptand hampers the formation of strong coordinative bonds. Consequently, square‐planar four‐membered [Bi_4_]^2−^ rings^15^ or [Bi_2_]^2−^ dumbbells^12^ are formed. Bi–Bi dumbbells can be seen as the result of a strong Peierls distortion within the planar polymer in **1**.

The [Bi_6_]^4−^ anion of compound **2** represents the heaviest homologue of this class of anions. The mechanism of formation of both new compounds is still unclear and needs further investigations, but their appearance reflects a preferred behavior of bismuthides to form rings or chains. Since both compounds were obtained under different reaction conditions employing different bismuthide precursors, we assume that there is an equilibrium of various Bi species in solution and only small differences may decide which species crystallizes.

## Conflict of interest

The authors declare no conflict of interest.

## Supporting information

As a service to our authors and readers, this journal provides supporting information supplied by the authors. Such materials are peer reviewed and may be re‐organized for online delivery, but are not copy‐edited or typeset. Technical support issues arising from supporting information (other than missing files) should be addressed to the authors.

Supplementary
