# Supplementary material for: Solvate‐Induced Semiconductor to Metal Transition: Flat 1/ [Bi1−] Zigzag Chains in Metallic KBi⋅NH3 versus 1/ [Bi1−] Helices in Semiconducting KBi
Source: Angew Chem Int Ed Engl. 2020 Mar 2;59(17):6800–5. doi: 10.1002/anie.201915735 (PMC7187340; doi:10.1002/anie.201915735)
Supplement: Supplementary file 1 — Supplementary [file ANIE-59-6800-s001.pdf]

Angewandte Chemie
Eine Zeitschrift der Gesellschaft Deutscher Chemiker

Supporting Information

Solvate-Induced Semiconductor to Metal Transition: Flat $^{1}_{\infty}[Bi^{1-}]$ Zigzag Chains in Metallic KBi·NH_3 versus $^{1}_{\infty}[Bi^{1-}]$ Helices in Semiconducting KBi

*Kerstin Mayer, Jasmin V. Dums, Christian B. Benda, Wilhelm Klein, and Thomas F. Fässler**

anie_201915735_sm_miscellaneous_information.pdf

Experimental Details

General. All reactions and manipulations were performed under a purified argon atmosphere using standard Schlenk and glove box techniques. The phases of nominal composition K_5Bi_4 and K_3Bi_2 were synthesized by heating (2 °C/min) of a stoichiometric mixture of both elements K (Merck, ≥ 98%) and Bi (99.99%, ChemPur) at 600 °C in a tantalum ampule for 5 d and slow cooling (1 °C/min) to room temperature. K_4Ge_9 and K_4Sn_9 were synthesized by heating (2°C/min) a stoichiometric mixture of K and Ge (99.999% ChemPur) or K and Sn (Merck, 99.98%) in a stainless-steel autoclave at 650 °C (K_4Ge_9) or 550 °C (K_4Sn_9) for 46 h and slow cooling (1°C/min) to room temperature. Liquid ammonia was stored over sodium metal for one day and freshly distilled before use. 18-Crown-6 (Merck) was purified by sublimation in vacuo. Ph_2Zn and $Mesnacnac_2Zn_2$ were synthesized according to literature methods.[1]

Synthesis of KBi · NH$_3$ (1). K_5Bi_4 (77 mg, 75 µmol), Ph_2Zn (16 mg, 75 µmol) and 18-crown-6 (36 mg, 135 µmol) were weighed out in a Schlenk tube inside a glove box. Approximately 2 mL of sodium-dried ammonia were condensed into this Schlenk tube yielding a greyish blue suspension. The Schlenk tube was stored at –70 °C. After four months several silvery plates suitable for single crystal diffractometry were obtained (yield approx. 15%). EDX results (calc. %): K 39.9% (50%), Bi 60.1% (50%).
Alternatively, compound **1** was formed during the reaction of K_3Bi_2 (160 mg, 300 µmol) with K_4Sn_9 (184 mg, 150 µmol) in liquid ammonia. Single crystals of compound **1** were found after 19 months (yield approx. 15%). As a side product crystals of $[K_5(OH)][Sn_9] \cdot 11\ NH_3$[2] were found.

Synthesis of K$_2$[K(18-crown-6)]$_2$[Bi$_6$] · 9 NH$_3$ (2). K_5Bi_4 (77 mg, 75 µmol), $Mesnacnac_2Zn_2$ (60 mg, 75 µmol) and 18-crown-6 (80 mg, 300 µmol) were weighed out in a Schlenk tube inside a glove box. Approximately 2 mL of sodium-dried ammonia were condensed into this Schlenk tube yielding a deep-blue suspension. The Schlenk tube was stored at –70 °C. After 35 months several black needles suitable for single crystal diffractometry were obtained (yield approx. 5%). EDX results (calc. %): K 43.7% (40%), Bi 56.3% (60%).
Alternatively, compound **2** was formed during the reaction of K_3Bi_2 (80 mg, 150 µmol), K_4Ge_9 (122 mg, 150 µmol) and 18-crown-6 (132 mg, 50 µmol) in liquid ammonia. Single crystals of compound **2** were found after 22 months (yield approx. 5%). As a side product crystals of $[K_5(OH)][Ge_9] \cdot 11\ NH_3$[3] were found.

Single-Crystal Structure Determination. Single crystals were fixed on a loop with perfluorinated ether and positioned in a 120 K cold N_2 stream. For single crystal X-ray diffraction data collection an Oxford-Xcalibur3 diffractometer (Mo-K_α radiation) was used. The structures were solved by Direct Methods and refined by full-matrix least-squares calculations against F^2 using SHELX-2014.[4] Non-hydrogen atoms were treated with anisotropic displacement parameters. Positions of the hydrogen atoms were

geometrically calculated and refined using a riding model. For compound **1** the unit cell metrics with all angles very close to 90° points to the orthorhombic crystal system. However, we could not find a way to describe the crystal structure in an orthorhombic space group, but instead we were successful in the monoclinic system with space group *Cm*. Our finding is supported by a check with the program Platon,[5] which does not detect any additional symmetry. Obviously, the positions of the ammonia molecules lower the apparent symmetry to the monoclinic crystal system. Table 1 comprises selected crystallographic data of compounds **1** and **2**. CSD 1901292 (**1**) and CCDC 1901293 (**2**) contain the supplementary crystallographic data for this paper. These data can be obtained free of charge from FIZ Karlsruhe via www.ccdc.cam.ac.uk/structures or from The Cambridge Crystallographic Data Centre via www.ccdc.cam.ac.uk/structures.

Table 1: Selected crystallographic data of the crystal structures of **1** and **2**.

Compound	**1**	**2**
formula	BiH_3KN	$C_{24}H_{75}Bi_6K_4N_9O_{12}$
fw (g·mol^{-1})	265.11	2092.21
space group	*Cm* (no. 8)	*Pmn*21 (no. 31)
a (Å)	5.2192(3)	24.8552(19)
b (Å)	15.3308(11)	8.0297(5)
c (Å)	5.4311(3)	14.5378(8)
α (deg)	90	90
β (deg)	90.004(6)	90
γ (deg)	90	90
V (Å^3)	434.57(5)	2901.5(3)
Z	4	2
T (K)	120(2)	120(2)
λ (Å)	0.71073	0.71073
ρ_{calcd} (g·cm^{-3})	4.052	2.395
μ (mm^{-1})	41.302	18.466
collected reflections	1306	23226
independent reflections	625	5789
R_{int}	0.0366	0.1452
parameters / restraints	31 / 0	260 / 0
R_1 [all data / $I > 2\sigma(I)$]	0.0210 / 0.0196	0.1037 / 0.0505
wR_2 [all data / $I > 2\sigma(I)$]	0.0468 / 0.0466	0.1061 / 0.0971
goodness of fit	1.085	0.830
max./min. diff. el. Density (e·Å^{-3})	1.00 / -1.39	2.23 / -1.61

Electron-Dispersive X-Ray (EDX) Analysis: Single crystals of all compounds were analyzed with a scanning electron microscope (JEOL 5900LV) equipped with an energy dispersive X-ray analyzer (Oxford Instruments).

Computational Details

Compound **1** and KBi were analyzed using the Crystal17 program package,[6] with exchange correlation hybrid functional after Perdew, Burke and Ernzerhof (PBE0)[7] and triple-zeta valence + polarization level basis set derived from the Karlsruhe basis sets for the elements Bi, N and H and a split-valence + polarization level basis set for K.[8] Basis set details are given below. Additionally, a generalized gradient approximation functional after Perdew, Burke and Ernzerhof (PBE)[7a] was used with the same basis sets. The starting geometry was taken from experimental data,[9] and all structures were fully optimized within the constraints imposed by the space group symmetry for both functionals. The band structure and density of states (DOS) were calculated. The nature of a stationary point on the potential energy surface could be confirmed by a frequency calculation for KBi, but for compound **1** a small imaginary frequency (40*i* cm^{-1}) was found. A rotation of the hydrogen atoms around the nitrogen is causing this frequency and vanished after symmetry reduction to *P1* and subsequently optimization. For data processing and visualization Jmol and VESTA 3 were used.[10]

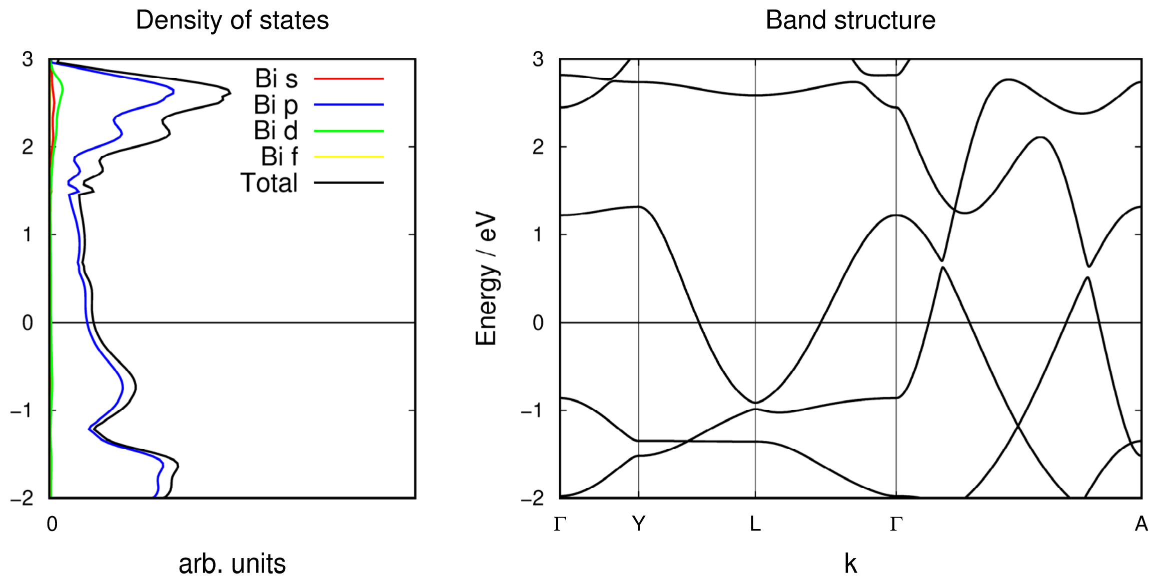

Figure S4: Orbital projected DOS and band structure of **1** showing a major precipitation of Bi *p* orbitals at the fermi level.

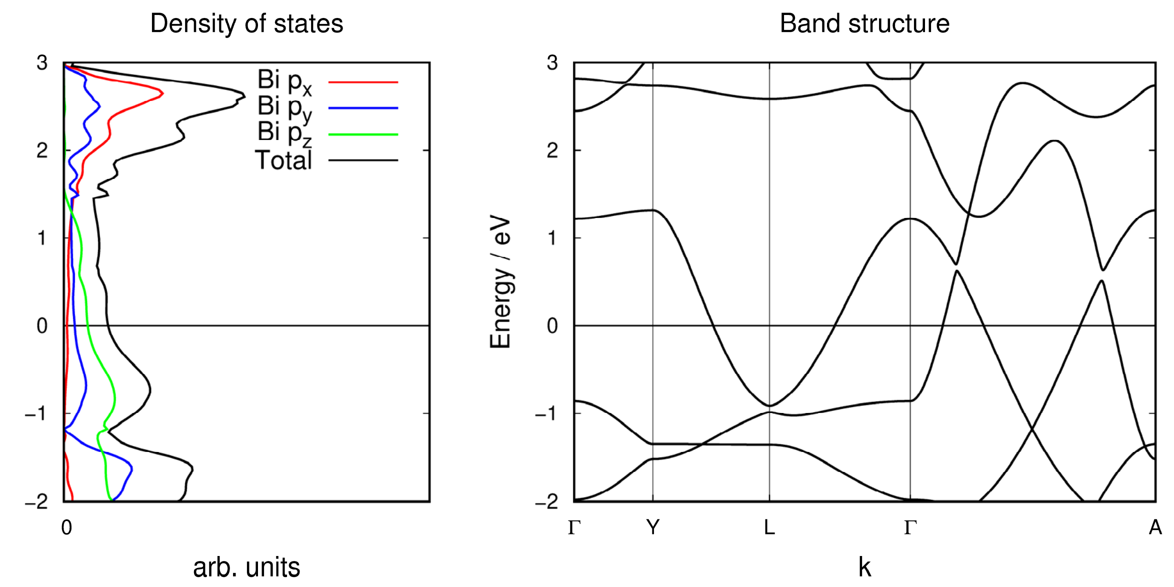

Figure S5: Orbital projected DOS and band structure of **1** showing a major precipitation of Bi p_z orbitals at the fermi level.

Table 2: Path of the band structure including labelling and coordinates in the Brillouin zone and direction in real space.

Point label (starting point)	Coordinates (starting point)	Point label (final point)	Coordinates (final point)	Direction in real space
Γ	0 0 0	Y	½ ½ 0	$\vec{b}$
Y	½ ½ 0	L	½ ½ ½	$\vec{c}$
L	½ ½ ½	Γ	0 0 0	n.a.
Γ	0 0 0	A	½ -½ 0	$\vec{a}$

Test 1: Detailed description of the used basis sets and basis set listening in CRYSTAL format.[18]

K: The SVP basis set was derived from the molecular Karlsruhe def-SVP basis set. The most diffuse *s* exponent of 0.012 was removed. The remaining outermost *s* exponent was increased from 0.029 to 0.14. The inner (3*s*) function was decontracted to (2*s*1*s*), and the exponent 0.282 was changed to 0.28. The most diffuse *p* exponent of 0.04 was increased to 0.14. The (3*p*) function was decontracted to (2*p*1*p*), and the exponent of 0.208 was increased to 0.28. The two outermost *s* and *p* functions were finally combined into two *sp*-type functions.

```
19 8
0 0 6 2.0 1.0
  31478.746764      0.39838653994E-02
  4726.8876066      0.30501759762E-01
  1075.4345353      0.15073752622
  303.39811023      0.51912939801
  98.327112831      1.0366957005
  33.636222177      0.76398963199
0 0 3 2.0 1.0
  65.639209962     -0.28242617106
  7.3162592218      1.6914935860
  2.8902580135      1.2965331953
0 0 2 2.0 1.0
  4.5459748965     -0.76343555273E-02
  .70404124062      0.25635718960E-01
0 1 1 1.0 1.0
  0.28              1.0 1.0
0 1 1 0.0 1.0
  0.14              1.0 1.0
0 2 5 6.0 1.0
  361.22492154      0.20906479823E-01
  84.670222166      0.15043641740
  26.469088236      0.55440061077
  9.2658077615      1.0409009991
  3.3423388293      0.67825341194
0 2 2 6.0 1.0
  1.5100876104      0.75248191146
  0.56568375163     1.3708585031
0 3 1 0.0 1.0
  0.353             1.0
```

Bi: We modified the molecular def2-TZVP basis set for periodic calculations by first removing the most diffuse *p* function with an exponent of 0.055, then fixing the exponents of the outermost *s* and *p* functions to 0.10, and finally reoptimizing the exponents of the other *s* and *p* functions in the valence space for the Bi atom in its ground state. The outermost *s* and *p* functions with an exponent of 0.1 were combined into one single *sp*-type function. The exponent of the outermost *d* function was increased from 0.14 to 0.165, and the steep *f* function with an exponent of 1.05 was removed. The basis set optimization was performed with the TURBOMOLE program package. The resulting energy loss w.r.t. the unmodified basis set is 3 mH.[11]

```
283 13
INPUT
23. 0 2 4 4 2 2
13.043090 283.264227 0
8.221682   62.471959 0
10.467777  72.001499 0
9.118901  144.002277 0
6.754791    5.007945 0
6.252592    9.991550 0
8.081474   36.396259 0
7.890595   54.597664 0
4.955556    9.984294 0
4.704559   14.981485 0
4.214546   13.713383 0
4.133400   18.194308 0
6.205709  -10.247443 0
6.227782  -12.955710 0
0 0 4 2.0 1.0
  716.41435310      0.31254307133E-03
  83.806059047      0.17624768946E-02
  21.116962853     -0.21910983437
  15.491448187      0.40411224931
0 0 2 2.0 1.0
  23.213322651     -0.68255758685E-01
  6.6412776932      0.97888046471
0 0 1 0.0 1.0
 1.7668753097       1.0000000000
0 0 1 0.0 1.0
 0.87636753866      1.0000000000
0 0 1 0.0 1.0
 0.26185055933      1.0000000000
0 1 1 0.0 1.0
 0.10               1.0 1.0
0 2 3 6.0 1.0
  15.249644669      0.74560356000
  14.846176053     -0.85578637338
  7.0636826784      0.40149159592
0 2 3 3.0 1.0
```

```
  2.5802708340      0.35542729633
  1.4990870077      0.63976991890
 0.75411119473      0.32332773839
0 2 1 0.0 1.0
  0.29617825843     1.0000000000
0 3 6 10.0 1.0
  66.404481948      0.38102878348E-03
  13.858426961      0.10746152442E-01
  7.0654519000     -0.71947646845E-01
  2.5252144035      0.26195974989
  1.3419585000      0.42594750000
 0.68340941000      0.33680325627
0 3 1 0.0 1.0
 0.32934755420      1.0000000000
0 3 1 0.0 1.0
 0.1646737771     1.0000000000
0 4 1 0.0 1.0
 0.31271            1.0000000000
```

N: We modified the molecular def-TZVP basis by removing the outermost *s* function, leaving still three *s* functions in the valence space. The exponents of the outermost *s* and *p* functions were set fixed to 0.22, and the exponents of the other *s* and *p* functions were optimized as discussed above for the SVP basis set. The resulting energy loss w.r.t. the unmodified basis set is 3 mH.

```
7 7
0 0 6 2.0 1.0
  19730.800647      0.21887984991E-03
  2957.8958745      0.16960708803E-02
  673.22133595      0.87954603538E-02
  190.68249494      0.35359382605E-01
  62.295441898      0.11095789217
  22.654161182      0.24982972552
0 0 2 2.0 1.0
  8.9809711129      0.40623896148
  3.6377863436      0.24338217176
0 0 1 0.0 1.0
 0.72643334701      1.0000000000
0 1 1 0.0 1.0
 0.22000000000      1.0 1.0
0 2 4 3.0 1.0
  63.222276180      0.55552416751E-02
  15.013291910      0.38052379723E-01
  4.6766636605      0.14953671029
  1.7814167689      0.34949305230
0 2 1 0.0 1.0
 0.68476710337      1.0000000000
0 3 1 0.0 1.0
 1.00000000000      1.0000000000
```

H: The def-TZVP basis set was modified by fixing the exponent of the outermost *s* function to 0.13 and reoptimizing the exponents of the other *s* functions in the valence space. The resulting energy loss w.r.t. the unmodified basis set is 0.15 mH.

```
1 4
0 0 3 1.0 1.0
  45.351741106      0.60251978000E-02
  6.8233001522      0.45021094000E-01
  1.5944879907      0.20189726000
0 0 1 0.0 1.0
 0.44546978735      1.00000000000
0 0 1 0.0 1.0
 0.13000000000      1.00000000000
0 2 1 0.0 1.0
 0.80               1.00000000000
```

References

[1] a) P. R. Markies, G. Schat, O. S. Akkerman, F. Bickelhaupt, W. J. J. Smeets, A. L. Spek, *Organometallics* **1990**, *9*, 2243-2247; b) S. Schulz, D. Schuchmann, U. Westphal, M. Bolte, *Organometallics* **2009**, *28*, 1590-1592.

[2] K. Mayer, J. Dums, W. Klein, T. F. Fässler, *Angew. Chem. Int. Ed.* **2017**, *56*, 15159-15163; *Angew. Chem.* **2017**, *129*, 15356-15361.

[3] C. B. Benda, H. He, W. Klein, M. Somer, T. F. Fässler, *Z. Anorg. Allg. Chem.* **2015**, *641*, 1080-1086.

[4] G. M. Sheldrick, *Acta Crystallogr., Sect. C: Struct. Chem.* **2015**, *71*, 3-8.

[5] A. L. Spek, Acta Crystallogr., *Sect. D: Biol. Crystallogr.* **2009**, *65*, 148-155.

[6] R. Dovesi, A. Erba, R. Orlando, C. M. Zicovich-Wilson, B. Civalleri, L. Maschio, M. Rerat, S. Casassa, J. Baima, S. Salustro, B. Kirtman. WIREs Comput Mol Sci. 8, e1360 (2018).; R. Dovesi, V. R. Saunders, C. Roetti, R. Orlando, C. M. Zicovich-Wilson, F. Pascale, B. Civalleri, K. Doll, N. M. Harrison, I. J. Bush, P. D'Arco, M. Llunell, M. Causà, Y. Noël, L. Maschio, A. Erba, M. Rerat, S. Casassa CRYSTAL17 User's Manual (University of Torino, Torino, 2017).

[7] a) J. P. Perdew, K. Burke, M. Ernzerhof, *Phys. Rev. Lett.* **1996**, *77*, 3865; b) C. Adamo, V. Barone, *J. Chem. Phys.* **1999**, *110*, 6158-6170.

[8] a) F. Weigend, M. Häser, H. Patzelt, R. Ahlrichs, *Chem. Phys. Lett.* **1998**, *294*, 143-152; b) F. Weigend, R. Ahlrichs, *Phys. Chem. Chem. Phys.* **2005**, *7*, 3297-3305; c) B. Metz, H. Stoll, M. Dolg, *J. Chem. Phys.* **2000**, *113*, 2563-2569; d) A. Karttunen, private communication.

[9] F. Emmerling, N. Längin, D. Petri, M. Kroeker, C. Röhr, *Z.Anorg. Allg. Chem.* **2004**, *630*, 171-178.

[10] a) *Jmol - An Open-Source Java Viewer for Chemical Structures in 3D,* The Jmol Team: 2017; b) K. Momma, F. I., Fujio, *J. Appl. Cryst.* **2011,** *44*, 1272-1276.

[11] TURBOMOLE V7.3 2018, a development of University of Karlsruhe and Forschungszentrum Karlsruhe GmbH, 1989-2007, TURBOMOLE GmbH, since 2007; available from http://www.turbomole.com.
